# Accessible and Validated Processing of SARS-CoV-2 from Wastewater

**DOI:** 10.1128/MRA.00174-21

**Published:** 2021-05-27

**Authors:** Nicole C. Rondeau, Oliver J. Rose, Lina A. Ariyan, Brian J. Mailloux, JJ L. Miranda

**Affiliations:** a Department of Biology, Barnard College, Columbia University, New York, New York, USA; b Office of Facilities Services, Barnard College, Columbia University, New York, New York, USA; c Environmental Science Department, Barnard College, Columbia University, New York, New York, USA; Indiana University, Bloomington

## Abstract

Severe acute respiratory syndrome coronavirus 2 (SARS-CoV-2), the etiological agent of coronavirus disease 2019 (COVID-19), is shed into wastewater. Accessible methods are necessary for processing samples in a myriad of contexts. We optimized a protocol for extracting viral RNA for downstream experiments. Our pipeline was validated with SARS-CoV-2 itself as a matrix recovery and quantitative measurement control.

## ANNOUNCEMENT

**FIG 1:**
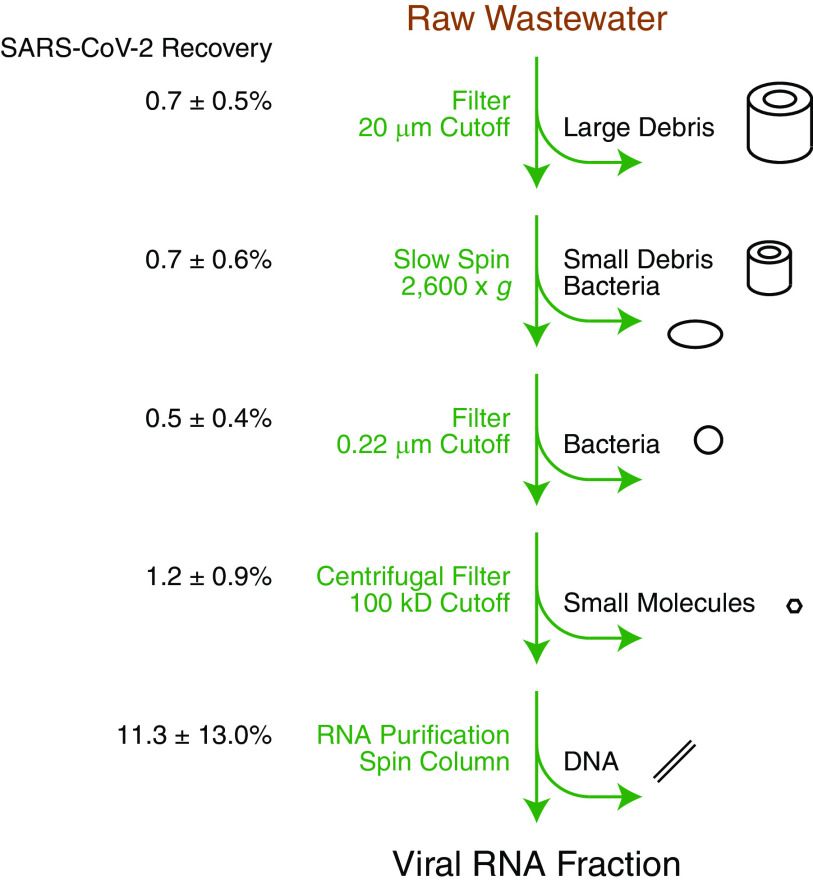
Accessible preparation of SARS-CoV-2 from wastewater for RNA analysis. Raw wastewater is processed with a 20-μm filter to remove large debris, slow centrifugation at 2,600 × *g* to remove small debris and bacteria, a 0.22-μm filter to remove bacteria, a 100-kDa centrifugal filter to remove small molecules, and spin column RNA purification to remove DNA. The resulting viral RNA fraction is suitable for subsequent PCR testing. Percentages on the left indicate the recovery of 10,000 SARS-CoV-2 genome equivalents/ml initial volume spiked in before the indicated processing steps. Numbers represent the mean and standard deviation of four independent biological replicates.

During the coronavirus disease 2019 (COVID-19) pandemic, stakeholders with a range of laboratory resources have started to monitor sewage for wastewater-based epidemiology (1). Many protocols for processing severe acute respiratory syndrome coronavirus 2 (SARS-CoV-2) exist (2), but not all are easily executed. We selectively incorporated parts of methods (3–5) that are accessible in resource-limited settings. Our new protocol (Fig. 1) emphasizes simplicity to allow adaptation to different infrastructures and team member training. No large equipment such as an ultracentrifuge is necessary. Multiple processing steps like membrane absorption are avoided. Disposables were leveraged to minimize cross-contamination.

We evaluated the efficiency of several processing steps and optimized an accessible protocol for the recovery of SARS-CoV-2 from wastewater samples. We obtained raw wastewater from Barnard College campus buildings with a Sentry ultra-compact composite sampler (N-Con Systems, Arnoldsville, GA). Specimens were pasteurized at 60°C for 1 h because of biosafety considerations (6) and processed in 40-ml batches. For validation, SARS-CoV-2 isolate USA-WA1/2020, heat inactivated (BEI Resources, Manassas, VA), was spiked into samples. Wastewater was filtered through a 20-μm Steriflip sterile centrifuge tube top filter unit (MilliporeSigma, Darmstadt, Germany), the filtrate was centrifuged at 2,600 × *g* for 10 min in a 50-ml conical tube, the supernatant was filtered through a 0.22-μm Steriflip-GP sterile centrifuge tube top filter unit (MilliporeSigma), and viruses were concentrated to <200 μl by repeated ultrafiltration of ∼10-ml aliquots in a 100-kDa-cutoff Amicon Ultra-15 centrifugal filter unit (MilliporeSigma) (Fig. 1). We purified RNA using a *Quick*-RNA miniprep kit (Zymo Research, Irvine, CA).

We measured SARS-CoV-2 using reverse transcription-quantitative PCR (RT-qPCR). Reactions included the 2019-nCoV_N1 forward primer GACCCCAAAATCAGCGAAAT, reverse primer TCTGGTTACTGCCAGTTGAATCTG, and probe 6-carboxyfluorescein (FAM)-ACCCCGCATTACGTTTGGTGGACC-BHQ1 from the 2019-nCoV RUO kit (Integrated DNA Technologies, Coralville, IA). The 2019-nCoV_RP forward primer AGATTTGGACCTGCGAGCG, reverse primer GAGCGGCTGTCTCCACAAGT, and probe FAM-TTCTGACCTGAAGGCTCTGCGCG-BHQ1, also from the 2019-nCoV RUO kit (Integrated DNA Technologies), were used as an invalid result extraction control (7). RT-qPCR was performed with the GoTaq probe 1-step RT-qPCR system (Promega, Madison, WI). We mixed 3.1 μl of nuclease-free water, 1.5 μl of combined primer/probe mix, 10 μl of GoTaq probe qPCR master mix with dUTP, 0.4 μl of GoScript RT mix for 1-step RT-qPCR, and 5 μl of RNA or a 1:10 dilution per reaction. A LightCycler 480 (Roche, Basel, Switzerland) performed the CDC-recommended amplification (7), as follows: 45°C for 2 min, 95°C for 2 min, 45 cycles of 95°C for 3 s with 55°C for 30 s, and 40°C for 30 s. Cycle threshold (*C_T_*) values were calculated by LightCycler 480 software (Roche). We determined a standard curve using quantitative PCR (qPCR) control RNA from heat-inactivated SARS-CoV-2 isolate USA-WA1/2020 (BEI Resources). Spike-in experiments were conducted only with samples that tested negative.

Our protocol was validated using SARS-CoV-2 instead of a substitute. This was not possible with methods (2) used early in the pandemic, prior to the availability of SARS-CoV-2 controls. The limit of detection was determined by spike-in experiments with 100 genome equivalents/ml, which consistently yielded positive-result *C_T_* values of <40 (Table 1). Observation of less than the expected ∼3.32-*C_T_* value increase in many RNA samples diluted 1:10 for RT-qPCR suggests the lingering presence of PCR inhibitors commonly found in wastewater. For more detailed evaluation of protocol efficiency, we spiked in 10,000 genome equivalents/ml initial volume at different steps. We used *C_T_* values from RNA samples diluted 1:10 and measured recovery of ∼1% of SARS-CoV-2 processed through the entire protocol. Parallel spike-in experiments at different steps identified concentration by centrifugal ultrafiltration as a critical procedure during which virus yield is reduced ∼10-fold (Fig. 1). We noted significant variability between samples, likely due to varied wastewater composition among sites and days. Overall, the protocol demonstrates sensitivity and efficiency suitable for accessible wastewater-based epidemiology.

**TABLE 1:** Detection of SARS-CoV-2 matrix recovery control in wastewater by RT-qPCR^*a*^

	Undiluted RNA (*C_T_*)		1:10 dilution of RNA (*C_T_*)	
Building	Replicate 1^*b*^	Replicate 2^*b*^	Replicate 1^*c*^	Replicate 2^*c*^
Dormitory A, replicate 1^*d*^	36.1	37.4	37.3	36.3
Dormitory A, replicate 2^*d*^	ND^*e*^	36.5	36.6	36.2
Dormitory B, replicate 1^*f*^	36.0	36.9	36.3	35.4
Dormitory B, replicate 2^*f*^	35.3	36.0	ND	ND
Dormitory B, replicate 3^*f*^	ND	ND	38.0	ND
Library	ND	34.8	ND	ND
Office	38.4	37.6	37.3	ND

a Heat-inactivated SARS-CoV-2 was added to samples at a concentration of 100 genome equivalents/ml.

b Technical replicate measurements of the same biological replicate.

c Technical replicate measurements of the same biological replicate.

d Independent biological replicates from the same sampling site, collected on different days.

*^e^* ND, not detected.

f Independent biological replicates from the same sampling site, collected on different days.

We hope that this simplified procedure provides a template for making experiments with or monitoring of SARS-CoV-2 in wastewater accessible to a broad audience. The protocol may also potentially be used to study other viruses or metagenomes.
