# Erratum for Rondeau et al., “Accessible and Validated Processing of SARS-CoV-2 from Wastewater”

**DOI:** 10.1128/mra.00236-23

**Published:** 2023-05-01

**Authors:** Nicole C. Rondeau, Oliver J. Rose, Lina A. Ariyan, Brian J. Mailloux, JJ L. Miranda

## ERRATUM

Volume 10, no. 21, e00174-21, 2021, https://journals.asm.org/doi/10.1128/MRA.00174-21. On page 2: the first amplification step, “45°C for 2 min” should read “45°C for 15 min”.

